# Prevalence of Albuminuria in Children Living in a Rural Agricultural and Fishing Subsistence Community in Lake Chapala, Mexico

**DOI:** 10.3390/ijerph14121577

**Published:** 2017-12-14

**Authors:** Felipe Lozano-Kasten, Erick Sierra-Diaz, Alfredo de Jesus Celis-de la Rosa, María Margarita Soto Gutiérrez, Alejandro Aarón Peregrina Lucano, Research Group on Social and Environmental Determinants in Childhood

**Affiliations:** Public Health Department, University of Guadalajara, C.P. 44340 Guadalajara, Jalisco, Mexico; f_lozano_k@hotmail.com (F.L.-K.); alfredo_celis@yahoo.com (A.d.J.C.-d.l.R.); marsoto_10@hotmail.com (M.M.S.G.); aapl69@hotmail.com (A.A.P.L.); iq_alfredo_co@yahoo.com.mx (R.G.S.E.D.C.)

**Keywords:** children, albuminuria, glomerular filtration rate, chronic kidney disease, rural community

## Abstract

The occurrence of Chronic Kidney Disease (CKD) of unknown etiology in autochthonous child populations residing along the Lake Chapala lakeshore is endemic (Jalisco, México). The objective of this study was to determine the prevalence of albuminuria in the pediatric population and to measure the glomerular filtration rate in children with two positive albuminuria tests. Urinary albumin was measured in 394 children. Subjects with two or more positive albuminuria test donated blood samples for the determination of serum biomarkers. From a rural community with 565 children under the age of 17 years, 394 (69.7%) participated with first morning urine samples. A total of 180 children were positive (with two or more positive albuminuria tests). The prevalence of albuminuria among the children participating in the study was 45.7%. Of the 180 children with persistent albuminuria, 160 (88.9%) were tested for serum creatinine, urea, and cystatin C. The 68.1% of the children studied, were found in stages 3a and 3b of the Kidney Disease Improving Global Outcomes (KDIGO) classification (mean glomerular filtration rate (GFR) 51.9 and 38.4 mL/min/1.73 m^2^ respectively). The lowest frequencies were for classifications 1 and 4. None of the subjects was classified as grade 5. The prevalence of albuminuria in children from this rural community is 3–5 times higher than reported in international literature. Regarding GFR, more than 50% of children studied are under 60 mL/min/1.73 m^2^. It is a priority to find the causes of albuminuria and CKD in this Mexican region.

## 1. Introduction

The occurrence of Chronic Kidney Disease (CKD) of unknown etiology in autochthonous child populations residing along the Lake Chapala lakeshore is endemic, mainly in the Poncitlán Municipality. CKD was mainly observed by population and health services at the beginning of the 1990s. It has been more than 20 years and the perception of the population is that CKD has increased. Part of the population and human right defenders affirm that the cause is found in the thermal water that is delivered to the communities from deep wells of the zone or in that extracted from Lake Chapala, although few inhabitants drink it (unpublished information, provided by University of Guadalajara academician). To date, there are no studies or data, to our knowledge, that could explain the high prevalence and/or causes of this decrease in glomerular filtration rate (GFR) in this population. Previous water-quality studies performed in this region by different of governmental and social agencies, in which heavy metals were sought, have not found that their results fell outside of the Mexican normative regulations.

At the beginning of 2016, the University of Guadalajara initiated an investigation project in the community of Agua Caliente localized on the shore of Lake Chapala. In this work, the authors included the determination of albumin in the urine of children aged less than six years who attended a kindergarten in the community. The prevalence of albuminuria as seen in these children was 18% (first morning urine sample).

Albuminuria is an early sign of possible damage to the kidney and is been used since the decade of the 1960s [1]. Since that time, measuring albuminuria has been used in the clinical field as a tool to measure kidney injury associated with diabetes mellitus and hypertension [2,3]. Once the relationship was established between albuminuria and kidney damage, methods for its quantification began to evolve, making them more rapid and precise [4].

Currently, albuminuria is recognized as a kidney and cardiovascular damage predictor, not only in patients with diabetes, but also in apparently healthy subjects. Its prevalence is reported as 7.2% in general population [5]. Most of the studies have been conducted in adults. However, in recent years, the trend has been aroused in detection at an early age due to future effects. In Japan, this method has been used since 1979 for the detection of kidney diseases in children (under the age of 19 years) [6], and other countries—such as Australia, England, Norway, the U.S., and Italy—have implemented this practice since the mid-1980s [7]. Information on prevalence is limited but is reported as approximately 7% [8]. Another study reported that the median ACR (albumin/creatinine ratio) of the random morning urine specimen for children <10 years was 2.91 vs. 2.28 mg/g (*p* < 0.001) for children ≥10 years. The median urinary albumin concentration for children <10 years was significantly higher than in children ≥10 years (2.53 vs. 2.09 µg/mL; *p* = 0.008). More recently, an Australian study considered 975 children aged 5–18 years. The overall prevalence of albuminuria, as defined by an ACR greater than 30 mg/g, was 12.8% (95% CI 9.9–15.6). In males, the frequency of albuminuria was 10.2% (95% CI 6.1–14.2) and in females, it was 15.5% (95% CI 10.7–20.3) [9,10,11].

In the available literature, there is enough information regarding albuminuria associated with diseases such as diabetes, hypertension, cardiopathies, and human immunodeficiency virus infection (HIV) in addition to different nephropathies in minors. The aims of this work are to describe the prevalence of albuminuria in general pediatric population from a rural community (Agua Caliente) in Jalisco, Mexico and to measure the glomerular filtration rate (GFR) (by means of the quantification of serum levels of creatinine, blood urea nitrogen (BUN), and cystatin C) in children identified with albuminuria.

## 2. Materials and Methods

In the community of Agua Caliente, Jalisco, a cross-sectional study was conducted after authorization of the Ethics Committee of the Public Health Department of the University of Guadalajara (DCSP/CEI/2016/10/176). As the first step, a census was taken of the population to identify the total number of inhabitants under 17 years who were invited to participate in the first stage of the project, which began in September 2016. The second step was to obtain sociodemographic and anthropometric data.

Informed consent was obtained from all individual participants included in the study (parents and children). Two first morning urine samples with an interval of 12 weeks were requested for participants. The samples were analyzed with reactive strips for the detection of human albumin Micral-Test^®^, (Roche Diagnostics GmbH, Mannheim, Germany). In those presenting two positive tests for albuminuria, blood tests were carried out for creatinine, urea, and cystatin C Diazyme’s Cystatin C Calibrator Set^®^ for immunoturbidometry assay, (Diazyme Laboratories, Inc., San Diego, CA, USA). All subjects in this group had blood pressure, weight, height, and body mass index (BMI) measures. From the data obtained, we calculated the GFR [12], which was adjusted to the body surface [13]. Based on the GFR, subjects were classified by grade of CKD according to guidelines of the Kidney Disease Improving Global Outcomes (KDIGO) clinical practice guidelines scale [14].

For data reporting, absolute frequencies, proportions, central trend measurements, confidence intervals (95%) were used. Data from the study were analyzed using Excel^®^ (Microsoft, Redmond, WA, USA) and SPSS^®^ (IBM, Armonk, NY, USA) software.

## 3. Results

Through a census carried out by academic personnel of the University of Guadalajara, it was determined that there is a population of 950 inhabitants, among whom 565 children under the age of 17 years were accounted for (Table 1). In this stage of the investigation, 394 (69.7%) children participated with two albuminuria tests, and some of them with more than two tests in order to confirm results. Children who were not included in the study were because their parents did not agree to participate. Seven children under one year with negative results were excluded. A total of 180 children were positive (with two or more positive albuminuria tests). The prevalence of albuminuria among the children participating in the study was 45.7% (95% CI: 40.7–50.8).

The Table 2 shows the data corresponding to height, weight, body mass index (BMI), and blood pressure of the two groups.

Distribution between positive and negative albuminuria frequency by age is depicted in Table 3.

Of the 180 children with persistent albuminuria, 20 were excluded by incomplete data and because their parents did not want to continue in the study. The remaining 160 (88.9%) donated blood for the determination of creatinine, urea, and cystatin C: 78 girls (42.4%) with an average age of 8.04 years (minimal age, three years; and maximal age, four years) and 82 boys with an average age of 7.46 years (minimal age, 1 year, maximal age, 17 years).

The result of the glomerular filtration rate is depicted in Table 4. The 68.1% of the children studied in this second step, were found in stages 3a and 3b of the KDIGO classification. The lowest frequencies were for classifications 1 and 4. None of the subjects was classified as grade 5.

In the group of children studied with positive albuminuria, none of the participants presented grade 5 CKD (Chronic Kidney Disease).

## 4. Discussion

Our results show an alarming prevalence of albuminuria in the children of this rural community. The observed prevalence was almost three to five times higher than international literature reports (45.7% vs. 2.2% to 12.8%) [8,9,10,11]. It is important to mention that 30.3% of the children from the community were not taken into account for the calculation of the prevalence. Even assuming that this 30.3% were negative for albuminuria, and were taken into account for the calculation, the prevalence would continue to be alarming (31.8%).

Regarding with glomerular filtration rate (GFR) obtained from the study subjects in stages 3a and 3b, the average calculated was 51.9 and 38.4 mL/min/1.73 m^2^, respectively. Based on KDIGO recommendations, in any child with GFR <60 mL/min/1.73 m^2^, a complete review of their past history and the clinical context should be considered in order to determine the cause(s) of kidney disease [14]. Even more important is the result obtained in 16 children between the ages of 2 and 10 years, in whom we calculated an average GFR of 27.6 mL/min/1.73 m^2^ (KDIGO 4) which may imply a potential health risk for these children due to the possibility of their advancing to stage 5.

With the results obtained to date, the usefulness is clear of the measurement of albumin in urine as a detection tool. Although the latter has been established as a method of scrutiny in Asiatic countries, international consensus does not yet exist on its use [15]. In the international literature, the association has been mentioned of albuminuria with environmental [16,17,18,19,20], demographic [21,22,23,24,25], infectious [26,27,28], non-infectious [29,30,31,32,33], and congenital [34,35,36,37,38,39,40] type factors.

In Australia, a study reported the prevalence of albuminuria of 7.3% in Aboriginal and non-Aboriginal children. The socioeconomic level and geographical situation were discarded as risk factors, after a two-year follow up [25]. From 2009–2011, in El Salvador, a study was carried on in 2115 children aged from 2–17 years, inhabitants in agricultural communities. The work reported prevalence of albuminuria up to 3.8 for both genders. It was also reported that the prevalence of CKD was up to 4.1 in women and of 3.6% in men. The glomerular filtration rate was carried out in 1960 of the participants and the authors did not utilize cystatin C for the calculation; the study results revealed that the average was greater than 140-mL/min/1.73 m^2^ [41]. The reported prevalence of albuminuria is found below the international mean, and even very much below that reported in our study (3.8% vs. 47.5%). It is clear the difference in regards to GFR (>140 vs. 48.7 mL/min/1.73 m^2^).

In Mexico, CKD in children is a problem that has been informed by different media. Multiple journalistic notes have reported health problems related with CKD. The Mexican states with the greatest number of notes of this type are Jalisco, Aguascalientes, and San Luis Potosí.

In 2008, Gongora, through the Health Institute of State of Aguacalientes, in collaboration with the British Columbia Centre for Disease Control, of Vancouver, Canada, carried out a study in which the authors evaluated 2712 schoolchildren, from 79 schools, with an average age of 9.23 years, finding 27.4% of the children with alterations in urine. A total of 19 children (0.7%) presented CKD, which established the prevalence of 700 cases in 100,000. Proteinuria was most frequent in boys in relation to girls (5.6% vs. 2.7%, respectively; *p* < 0.00001). With regards to CKD stages, stage II (47.4%) was the most frequent, followed by stages III (26.3%), I (15.7%), and IV and V (5.3% each) [42].

At mid-2016, Cárdenas published a study in which was related exposure to environmental contaminants and CKD in children in San Luis Potosí. The work was carried out in a community with 10,383 inhabitants. The community members noticed an unusual number of young adults with kidney diseases in the area. Data from the Ministry of Health of the State of San Luis Potosí reported that genitourinary diseases comprised the third cause of death in the region. These data also indicated that 25% of the population was found in a situation of extreme poverty, 22.6% did not have running water in their homes, and 66% lacked access to health services. The study design was transversal, which included 107 girls and boys between the ages of 5 and 12 years, who responded to questionnaires and analyses of heavy metals such as arsenic, cadmium, chromium (Cr), and fluorine in urine, in addition to lead in serum lead. These results were related to other tests for determination of kidney function (Cr and GFR). The GFR mean was 93.4 and 84 mL/min for girls and boys, respectively. The novelty of the work lies in the association of the urinary concentrations of heavy metals outside of the normative regulations, with non-conventional kidney markers (KIM-1, NGAL, and miR21) [43].

Regarding the Community of Agua Caliente, Poncitlán, Jalisco, health problems have been more noticeable over last months. To date, to our knowledge, there are no data or studies that could explain the high prevalence of and/or causes of this increase in CKD in this population and in some others in the same area. However, since 2012, the University of Guadalajara has conducted field research in the area that describes the environmental problems that affect the population health [44].

The community of Agua Caliente is a locality at a distance, in which nearly 100% of the inhabitants have similar life conditions, highlighting the situation of extreme poverty. Among the shared problems, we find some uses and customs, as well as the lack of prevention and health-promotion strategies. The Agua Caliente community has a kindergarten, a primary school, a secondary school, and a primary-level health center with a morning schedule. One of the main economic activities of the community is agriculture (37.9%), specifically the cultivation of vegetable pears. The remainder of the population is dedicated to activities such as fishing, construction, and household tasks. The principal food sources are the fish that are directly extracted from Lake Chapala and the consumption of fruits and vegetables cultivated in the community. Other products (milk, rice, beans, and canned products) are obtained from stores in the community or through government aid. The water that the inhabitants consume is bottled and derives from local bottlers. For food preparation, 94% of households cook with firewood. The community does not have trash collection services; thus, in 94% of homes, trash is burned on a daily basis.

In addition to smoke pollution, The University of Guadalajara researchers detected other problems, such as the non-monitored use of agrochemicals, alcoholism, extreme poverty, child malnutrition, intestinal parasitosis, and gender inequity. All of these factors put the health of the population at risk, especially in children.

These antecedents could suggest some hypotheses with respect to the cause of CKD in children, and while these studies do not coincide in the variables measured, it is evident that there is a problem that is not solved to date, it is even more important to know the situation of hundreds of communities throughout the Mexican Republic that entertain the same conditions, or even poorer ones, that those of the population of Agua Caliente.

One of the limitations of our study is the lack of non-exposed population, that is, children who do not live in the community, in order to compare the results. The community’s characteristics are very particular. The children studied share many characteristics that differ in great part with other populations. A certain advantage is that not including children from other places allows us to minimize confounding factors. It is noteworthy that we are planning to implement the same investigation model in other communities in the state of Jalisco that have similar problems but that are found at a great distance from Lake Chapala and that additionally have other uses and customs.

## 5. Conclusions

At present, this is only an initial report of a cohort study, a project of great importance for the University of Guadalajara in association with New York University and the Government of the State of Jalisco. It is impossible to ignore the alarming nature of the data because there exists the probability that other communities in Mexico have the same problem. The principal perspective is to identify and define the causal (probably multicausal) model in order to collaborate with an efficient medical design and to diminish the prevalence of CKD in the community and to assess its external validity to apply it in other communities localized at a distance with similar problems. However, much remains to be investigated in order to arrive at the core of the problem and to find a solution to it. Willingness of the part of University of Guadalajara authorities and the participation of the population in collaborating with the study comprise the greatest strengths of our investigation. It is impossible to avoid mentioning the strength of academicians, researchers, and university students who actively participate in the investigation, all with the purpose of creating and collecting knowledge on this phenomenon, which additionally affects an important part of the vulnerable population, one of the most important public health problems in Mexico.

## Figures and Tables

**Table 1:** Frequencies of albuminuria in children studied in the community of Agua Caliente, Jalisco.

Cases	Frequency	Percentage (95% CI *)
Participating Children	394	69.7
Persistent albuminuria	180	45.7 (40.7–50.8)
Negative albuminuria	214	54.3 (49.3–59.3)
Non-participants	171	30.3
Total	565	100.0

* 95% Confidence Interval estimated by Fisher Exact at OpenEpi.com.

**Table 2:** Data related to height, weight, BMI, and blood pressure in children with albuminuria positive and negative test.

Age Group (Years)	Variable	Albuminuria	*n*	Mean	SD	95% CI ^A^	*p* ^B^
1–4	Weight (kg)	No	32	13.95	3.22	1279–1511	0.49
		Yes	27	14.84	2.22	1396–1572	
	Height (cm)	No	32	94.25	11.65	9005–9845	0.61
		Yes	27	96.48	7.06	9369–9927	
	BMI	No	32	15.63	2.19	1484–1642	0.61
		Yes	27	15.92	1.53	1531–1653	
	Systolic BP (mmHg)	No	32	87.25	8.54	8417–9033	0.87
		Yes	27	88.44	14.37	8276–9412	
	Diastolic BP (mmHg)	No	32	53.34	7.64	5059–5609	0.83
		Yes	27	54.52	10.90	5021–5883	
5–9	Weight (kg)	No	100	22.28	4.24	2144–2312	0.16
		Yes	84	21.57	4.91	2050–2264	
	Height (cm)	No	100	118.87	8.51	11,718–12,056	0.72
		Yes	84	118.43	10.28	11,620–12,066	
	BMI	No	100	15.65	1.48	1536–1594	0.01
		Yes	84	15.18	1.92	1476–1560	
	Systolic BP (mmHg)	No	100	89.52	11.06	8733–9171	0.48
		Yes	84	91.40	9.14	8942–9338	
	Diastolic BP (mmHg)	No	100	55.36	7.54	5386–5686	0.94
		Yes	84	55.44	7.88	5373–5715	
10–14	Weight (kg)	No	76	36.06	7.80	3428–3784	0.38
		Yes	46	34.90	8.83	3228–3752	
	Height (cm)	No	76	139.83	18.10	13,569–14,397	0.51
		Yes	46	140.05	8.85	13,742–14,268	
	BMI	No	76	17.73	2.38	1719–1827	0.45
		Yes	46	17.50	20.61	1138–2362	
	Systolic BP (mmHg)	No	76	113.63	98.35	9116–13,610	0.73
		Yes	46	100.78	12.04	9720–10,436	
	Diastolic BP (mmHg)	No	76	65.87	9.76	6364–6810	0.27
		Yes	46	64.09	9.82	6117–6701	
15–17	Weight (kg)	No	5	53.62	8.62	4292–6432	0.57
		Yes	3	57.3	7.59	3845–7615	
	Height (cm)	No	6	153.17	15.46	13,695–16,939	0.12
		Yes	3	167.47	8.95	14,524–18,970	
	BMI	No	5	21.26	4.17	1608–2644	0.65
		Yes	3	20.40	1.73	1610–2470	
	Systolic BP (mmHg)	No	6	109.00	10.68	9779–12,021	0.44
		Yes	3	116.00	3.00	10,855–12,345	
	Diastolic BP (mmHg)	No	6	66.17	11.02	5461–7773	0.04
		Yes	3	79.67	3.06	7207–8727	

^A^ Mean confidence interval. ^B^ *p* values correspond to differences between positive and negative albuminuria groups estimated by Mann–Whitney U test.

**Table 3:** Distribution of groups by age.

Age Group	Negative Albuminuria	Positive Albuminuria	95% CI * for Positive Albuminuria
1–4 years	32 (54.2%)	27 (45.8%)	32.7–59.3
5–9 years	100 (54.3%)	84 (45.7%)	38.3–53.1
10–14 years	76 (62.3%)	46 (37.7%)	29.0–46.9
15–17 years	6 (66.7%)	3 (33.3%)	7.5–70.0
Total	214 (57.2%)	160 (42.8%)	37.7–48.0

* 95% Confidence Interval estimated by Fisher Exact at OpenEpi.com.

**Table 4:** Frequency and distribution of the Glomerular Filtration Rates (GFR) in the different age groups.

Age Group	GFR G1	GFR G2	GFR G3a	GFR G3b	GFR G4	Total
<1 year	0	0	0	0	0	0
1–4 years	0	1	1	14	11	27
5–9 years	0	7	31	42	4	84
10–14 years	2	23	15	5	1	46
15–17 years	0	2	1	0	0	3
Total	2	33	48	61	16	160
	(1.3%)	(20.6%)	(30%)	(38.1%)	(10%)	(100%)
Prevalence (95% CI) * [*n* = 374]	0.53 (0.07–1.9)	8.8 (6.2–12.1)	12.8 (9.6–16.7)	16.3 (12.7–20.5)	4.3 (2.5–6.9)	42.8 (37.7–48.0)

* 95% Confidence Interval estimated by Fisher Exact at OpenEpi.com.
